# Planthopper-Secreted Salivary Disulfide Isomerase Activates Immune Responses in Plants

**DOI:** 10.3389/fpls.2020.622513

**Published:** 2021-01-18

**Authors:** Jianmei Fu, Yu Shi, Lu Wang, Hao Zhang, Jing Li, Jichao Fang, Rui Ji

**Affiliations:** ^1^Institute of Plant Protection, Jiangsu Academy of Agricultural Sciences, Jiangsu Key Laboratory for Food and Safety-State Key Laboratory Cultivation Base of Ministry of Science and Technology, Nanjing, China; ^2^College of Plant Protection, Nanjing Agricultural University, Nanjing, China; ^3^School of the Environment and Safety Engineering, Jiangsu University, Zhenjiang, China

**Keywords:** small brown planthopper, salivary elicitor, plant defense, reactive oxygen species, jasmonic acid signaling pathway

## Abstract

The small brown planthopper (*Laodelphax striatellus*; SBPH) is a piercing-sucking insect that secretes salivary proteins into its plant host during feeding. However, the mechanisms by which these salivary proteins regulate plant defense responses remain poorly understood. Here, we identified the disulfide isomerase (LsPDI1) in the SBPH salivary proteome. LsPDI1 was highly expressed in the SBPH salivary glands and secreted into rice plants during feeding. Transient *in planta* LsPDI1 expression in the absence of signal peptide induced reactive oxygen species (ROS) burst, cell death, callose deposition, and jasmonic acid (JA) signaling pathway. Deletion mutant analysis revealed that either the a-b-b’ or the b-b’-a’ domains in *LsPDI1* are required to induce cell death in plants. LsPDI1 and its orthologs were highly conserved among various planthopper species and strongly induced ROS burst and cell death in plants. Transient in *Nicotiana benthamiana* LsPDI1 expression impaired the performance of *Spodoptera frugiperda* and *Myzus persicae* on host plants. Hence, LsPDI1 is an important salivary elicitor that enhances plant resistance to insects by inducing the calcium, ROS, and JA signaling pathways. The findings of this study provide novel insights into the molecular mechanisms underlying plant-insect interactions.

## Introduction

Plants and herbivores have co-evolved and have been engaged in a long-term arms race. Piercing-sucking insects commonly inject salivary proteins into plant cells during feeding (Sōgawa, 1982; Alon et al., 2013; Elzinga et al., 2014; Elodie et al., 2015; Villarroel et al., 2016; Xu et al., 2018; Huang et al., 2019). In response, plant pattern recognition receptors perceive salivary proteins and activate complex defense responses, including calcium influx, mitogen-activated protein kinase (MAPK) cascades, reactive oxygen species (ROS), and jasmonic acid (JA), salicylic acid (SA), and ethylene (ET) signaling (Wu and Baldwin, 2010a). There is mounting recent evidences have demonstrated that salivary elicitors trigger host plants defenses. The salivary proteins Mp10, Mp42, Mp56, Mp57, and Mp58 secreted by aphids inhibit insect reproduction by activating plant defense responses (Bos et al., 2010; Elzinga et al., 2014). The salivary protein NlMLP, Nl12, Nl16, Nl28, Nl43, and NlSP1 secreted by brown planthopper (*Nilaparvata lugens*, BPH) are recognized by host plants and elicit defense responses in them (Shangguan et al., 2017; Rao et al., 2019; Huang et al., 2020). However, other than in aphids, there is limited information about the salivary secretions of other insect herbivores that elicit plant defense responses.

Protein disulfide isomerases (PDIs) are ubiquitous multifunctional enzymes. These members of the thioredoxin superfamily occur in eukaryotic organisms. Classical PDIs are highly conserved and consist of the *N*-terminal signal peptide, the a, b, b’, and a’ domains, and a *C*-terminal ER retention signal (D’Aloisio et al., 2010; Shergalis and Neamati, 2016). The structures of the catalytic domains a and a’ resemble that of thioredoxin, with the conserved CGHC active site which is vital for the oxidoreductase function of PDIs (Appenzeller-Herzog and Ellgaard, 2008). The non-catalytic domains b and b’ can recognize substrates with the assistance of the a’ domain (Tian et al., 2006). PDIs function in oxidation, reduction and isomerization, and as chaperone and anti-chaperones (Shergalis and Neamati, 2016). PDIs catalyze the oxidative folding of new peptides in the endoplasmic reticulum, stabilize protein conformations, and perform other cellular functions. Further, recent evidences indicate that PDIs participate in pathogenesis (Stolf et al., 2011; Meng et al., 2015). Thus, for example, Meng et al. (2015) reported that PpPDI1 is a putative *Phytophthora parasitica* (*P. parasitica*) virulence factor and promotes plant infection. Proteomics disclosed several PDIs in herbivorous insect saliva (Carolan et al., 2011; Van Bel and Will, 2016; Huang et al., 2018; Miao et al., 2018; Rao et al., 2019). However, the mechanisms by which PDIs modulate plant host defense responses remain unknown.

The small brown planthopper (*Laodelphax striatellus*, SBPH) has mouthparts that pierce and suck phloem. It is a major agricultural pest in Asia and feeds on rice, barley, wheat, corn, and other gramineous plants. SBPH has wide host selectivity, multiple generations, and strong reproductive capacity. It can also transmit plant viral pathogens, causing serious annual crop yield losses of the aforementioned crops (Otuka et al., 2010). The components of SBPH saliva have been identified. However, to date, it has only been confirmed that salivary DNase II from SBPH suppresses H_2_O_2_ accumulation in host rice by degrading extracellular DNA in rice (Huang et al., 2019); little is known about others biochemical mechanisms in the plant-insect interaction.

Here, we used *Agrobacterium*-mediated transient expression to screen SBPH salivary proteins that induce cell death in *Nicotiana benthamiana* (*N. benthamiana*) leaves. LsPDI1, a typical PDIs-like protein that acts as a salivary elicitor in plant-insect interactions, was newly discovered. It was secreted into host plant cells and strongly induced cell death, possibly via the calcium signal transduction pathway and ROS and defense responses via the jasmonic acid (JA) signaling pathway. Its orthologs NlPDI1 and SfPDI1 from the BPH or white-backed planthopper (*Sogatella furcifera*, WBPH) are highly conserved and also induce ROS burst and cell death in host plants. When *LsPDI1* was transiently expressed in plants, it impaired the performance of the chewing insect *Spodoptera frugiperda* and the sap-sucking insect *Myzus persicae*. The findings of this study may help elucidate molecular-level plant-insect interactions and develop novel strategies to control herbivorous pest insects.

## Materials and Methods

### Insects and Plants

Small brown planthopper were reared on the seedlings of the susceptible rice cultivar Taichung Native 1 (TN1) under constant temperature of 26 ± 1°C and a 16 h/8 h light/dark cycle. Green peach aphids (*Myzus persicae*) were maintained on *N. benthamiana* under controlled environmental conditions (18 ± 1°C; 16 h/8 h light/dark cycle). *Spodoptera frugiperda* larvae were maintained on an artificial diet under constant temperature of 26 ± 1°C and a 16 h/8 h light/dark cycle.

*Nicotiana benthamiana* were cultivated in a greenhouse under a 16 h/8 h light-dark cycle. The daytime and nighttime temperature ranges were 22–25°C and 18–22°C, respectively. After 4–6 weeks (five-leaf stage), the plants were used in an *Agrobacterium* GV3101-mediated transient transformation experiment.

### Watery Salivary Protein Collection and High-Throughput Sequencing (Shotgun LC-MS/MS)

Watery salivary protein from fifth-instar SBPH nymphs was collected and concentrated as previously described (Huang et al., 2018). Briefly, ∼100–150 nymphs were placed in a glass tube containing 0.3 mL of 2.5% (w/v) sucrose. The tube was sealed with two Parafilm^TM^ (Bemis Co., Neenah, WI, United States) strips. After 24 h, the sucrose solution was collected and concentrated with a 3 kDa Millipore series (EMD Millipore, Billerica, MA, United States), trichloroacetic acid (TCA), and acetone. To avoid microsite differences, three replicates were performed, each containing of ∼8,000 individuals from ∼53–80 glass tubes containing ∼100–150 nymphs per tube.

Concentrated samples were sent to Shanghai Applied Protein Technology Co., Ltd. (Shanghai, China) for enzymolysis by filter-aided sample preparation (Wisniewski et al., 2009). NanoLC-MS/MS analysis of the watery saliva proteins was performed as previously described (Huang et al., 2018).

### Spatiotemporal Expression of Candidate Salivary Protein Genes Measured by RT-qPCR

Whole *L. striatellus* RNA samples were prepared at the egg, 1st–5th-instar nymph, and newly emerged brachypterous male and female adult stages. RNA samples were also prepared for the heads without the salivary glands, guts, ovaries, and fat bodies dissected from brachypterous female adults. Total RNA was extracted using a SV (spin or vacuum purification protocol) total RNA isolation kit (Promega, Madison, WI, United States) according to the manufacturer’s instructions. To minimize the influence of individual differences, ten samples per replicate were pooled and five replicate pots were prepared. One microgram RNA was reverse-transcribed into cDNA using a PrimeScript RT reagent kit with gDNA Eraser (RR047A; TaKaRa Bio Inc., Kusatsu, Shiga, Japan). Real-time quantitative polymerase chain reaction (RT-qPCR) was performed using a TB Green^TM^ Premix Ex Taq^TM^ kit (TaKaRa Bio Inc., Kusatsu, Shiga, Japan) in a LightCycler^®^ 480 II qPCR (Roche Diagnostics, Basel, Switzerland). The primers were designed with Primer Premier v. 5.0 (Supplementary Table 1). The housekeeping gene β*-actin* was used to normalize cDNA concentrations. Relative expression ratios were calculated by the Pfaffl method (Pfaffl, 2001).

### Antibody Preparation and Detection of the LsPDI1 Protein Secreted Into Rice Plants Using Western Blotting

Rabbit anti-LsPDI1 polyclonal antibodies against a specific peptide (KIILFKQFDEGKAIFE) was prepared by Genscript (Nanjing, China). Briefly, the specific peptide was synthesized for rabbit inoculation, and the immune response was validated by ELISA and western blotting to check the specificity of this antibody.

The salivary glands of 100 fifth-instar SBPH nymphs were collected and homogenized in 1 mL phosphate-buffered saline (PBS). The extract was centrifuged at 12,000 × *g* and 4°C for 5 min and the supernatant was collected. Rice stems were individually confined in ventilated glass cylinders, and then 200 fifth-instar SBPH nymphs were released into each cylinder and removed after 2 days. Rice stems without insects served as controls. The outer three leaf sheaths were harvested from each rice stem and pulverized with liquid nitrogen. Then, 2 mL NP40 buffer (Beyotime Institute of Biotechnology, Jiangsu, China) was added and the suspension was mixed at 4°C for 20 min. The samples were centrifuged twice at 15,200 × *g* and 4°C for 5 min. The supernatants were collected and concentrated to 200 μL using a YM-10 Microcon centrifugal filter device (EMD Millipore, Billerica, MA, United States).

Western blotting was performed as previously described (Fu and Liu, 2020). Briefly, 50 μL of supernatant per sample (∼400 μg) was combined with 5 × SDS buffer, and the mixture was heated to 95°C for 10 min. Proteins were isolated using 4–20% prefabricated SDS-PAGE gels (Bio-Rad Laboratories, Hercules, CA, United States) in 1 × running buffer (Tris 30.2 g, glycine 188 g, SDS 10 g, volume to 1 L with ultrapure water). The proteins were then transferred to a polyvinylidene fluoride (PVDF) membrane using a Tris-Gly membrane transfer system (Tris 3.94 g, glycine 18.72 g, methanol 195 mL, ultrapure water 1105 mL; 40°C; 40 V; 14–18 h). The membrane was thereafter blocked by immersion in 3% (v/v) bovine serum albumin and shaken at 80 rpm and 30°C for 2 h. The blocked membrane was washed with Tris-buffered saline (TBS) for 3–5 min, and then subjected to immunoblotting in an iBind western blot device (Invitrogen, Carlsbad, CA, United States) with 1 × iBind Flex solution (100 × additive, iBind Flex 5 × buffer, and pure water). The anti-LsPDI1 (1:500), sheep anti-rat (1:2,000; Abcam, Cambridge, United Kingdom) and Marker HRP (1:1,000; Bio-Rad Laboratories, California, United States) were used as primary antibodies and secondary antibodies, respectively. The membrane was incubated with enhanced chemiluminescence (ECL) color developer and enhancement solution (1:1 mixture) for 5 min, and the colored bands that developed on the membrane were viewed using a VersaDoc imaging system (Bio-Rad Laboratories, Hercules, CA, United States).

### Rice Protoplast Isolation and Transfection

Rice protoplasts were isolated as previously described (Zhang et al., 2011). The *LsPDI1* coding region was cloned from cDNA reverse-transcribed from SBPH salivary gland RNA and inserted into a *Pbinplus-GFP* vector (*GFP*). Sequencing was detected and polyethylene glycol (PEG)-mediated recombinant plasmid *LsPDI1-GFP* transformation into rice protoplasts was performed as previously described (Zhang et al., 2011). The GFP (488 nm) and mCherry (561 nm) fluorescence signals were observed under a Zeiss LSM750 confocal laser-scanning microscope (Carl Zeiss AG, Oberkochen, Germany).

### Transient Expression System Development and Defense-Related Parameter Determination

The recombinant plasmid *LsPDI1-GFP* was transformed into *Agrobacterium* strain GV3101. The latter was resuspended in an infiltration buffer (10 mM MgCl_2_, 500 mM MES, and 100 mM acetosyringone) and infiltrated into *N. benthamiana* leaves at OD_600_ = 0.4. ROS were identified by 3,3’-diaminobenzidine (DAB) staining as previously described (Wang et al., 2017), with certain modifications. Briefly, *N. benthamiana* leaves were immersed in phosphate buffer (0.02 M; pH 7.0) containing 1 mg/mL DAB and vacuumed in the dark for 10 min. The leaves were incubated overnight, decolorized with 2.5% (v/v) trichloroacetaldehyde until transparent, and photographed.

Cell death was evaluated by measuring ion leakage and Trypan blue staining in *N. benthamiana* leaf disks (Huang et al., 2020), with certain modifications. Briefly, 15 *N. benthamiana* leaf disks were excised with a punch (*r* = 12 mm) and placed in 20 mL distilled water for 30 min until they were completely imbibed. They were then stored at 25°C for 4 h and their initial conductivity S_1_ was measured with a conductivity meter (Mettler Toledo, Greifensee, Switzerland). They were then sealed and transferred to a boiling water bath for 10 min. S_2_ conductivity was measured until the liquid cooled. Distilled water conductivity was designated as S_0_. Relative conductivity was calculated as follows:

(1) $$100\times \left(S_{1}-S_{0}\right)/\left(S_{2}-S_{0}\right)$$

The *N. benthamiana* leaves were infused with Trypan blue solution by vacuuming and immersed in a boiling water bath until they turned blue. The leaves were decolorized with 2.5% (v/v) trichloroacetaldehyde until transparent and were then photographed.

Rice protoplast viability was evaluated by fluorescein diacetate (FDA) staining and luciferin/luciferase reporter (LUC) assay as previously described, with certain modifications (Zhang et al., 2011; Zhao et al., 2016). For the FDA staining, 0.5 mL protoplast suspension was transferred to 1.5 mL EP, and sufficient FDA solution was added to make a final concentration of 0.01% (v/v). The suspension was gently mixed and stored in the dark at 25°C for 5 min. Green fluorescence (488 nm) was observed and photographed under a Zeiss LSM750 confocal laser-scanning microscope (Carl Zeiss AG, Oberkochen, Germany) after transient LsPDI1-GFP expression for 16 h. The protoplasts were scored under a fluorescence microscope for ≥ 10 randomly selected fields. For the LUC assay, plasmids containing the LUC reporter gene were co-transfected by PEG-CaCl_2_ with plasmids containing *LsPDI1-GFP* or *GFP* in rice protoplasts. After 40 h, the LUC activity was measured in a LUC assay system (Promega, Madison, WI, United States).

### Cell Death Inhibition Assays Using LaCl_3_ and *Bcl-XL*

The effects of the calcium channel inhibitor LaCl_3_ and the human antiapoptotic gene *Bcl-XL* (accession No. Z23115.1) on cell death were determined in the dark as previously described (Chen et al., 2013). Briefly, *Agrobacterium* or rice protoplast suspensions containing *LsPDI1-GFP* or *GFP* plasmids were suspended in 1 mM LaCl_3_ and transformed into *N. benthamiana* or rice protoplasts. *Bcl-XL* was synthesized and constructed by homologous recombination into a GFP vector. *Bcl-XL-GFP* was co-transfected with other plasmids containing *LsPDI1-GFP* or *GFP* into *N. benthamiana* or rice protoplasts. *Bcl-XL* expression vectors were expressed in *N. benthamiana* leaves for 24 h before introducing *LsPDI1-GFP* or *GFP*. DAB staining, Trypan blue staining, and relative LUC fluorescence activity were performed at 12, 24, and 40 h, respectively, as previously described.

### Callose Deposition and Plant Defense-Related Gene Expression Assays in *N. benthamiana*

Infiltrated *N. benthamiana* leaf disks were subjected to 0.05% (w/v) aniline blue staining and trichloroacetic acid (TCA; 2.5 g/mL) decolorization. Callose deposition was visualized as previously described (Wang et al., 2017), with certain modifications. The leaf disks were observed and photographed after 24 h under a Zeiss LSM750 confocal laser-scanning microscope (Carl Zeiss AG, Oberkochen, Germany) fitted with a UV filter. Fluorescence was quantified with ImageJ v. 1.8.0 (NIH, Bethesda, MD, United States). Each sample was scored for ≥ 10 randomly selected microscopic fields.

Expression levels of the defense-related genes induced by LsPDI1-GFP were detected by RT-qPCR. Total RNA was extracted from *N. benthamiana* leaves infiltrated with *Agrobacterium* strain GV3101 at 24 and 48 h using the RNeasy plant mini kit (Qiagen, Hilden, Germany) or from PEG-transfected rice protoplasts at 12 and 24 h using the EASYspin tissue/cell RNA rapid extraction kit (Yuanpinghao Biotech, Beijing, China). The cDNA was synthesized and RT-qPCR was performed to determine the gene expression levels. Five independent biological replicates were performed per treatment and three samples were pooled. Primers were designed with Primer Premier v. 5.0 (Supplementary Table 1). β-actin and *GAPDH* were the housekeeping genes. The relative quantitative method (2^–ΔΔ*Ct*^) evaluated variations in expression level among samples (Livak and Schmittgen, 2001).

The samples were harvested from *N. benthamiana* leaves infiltrated with *LsPDI1-GFP* and *GFP Agrobacterium* strain GV3101 at 24 and 48 h after the start of the treatment. Samples were ground in liquid nitrogen, and SA, JA, and jasmonic acid-isoleucine (JA-Ile) were extracted with ethyl acetate spiked with labeled internal standards (^2^D_4_-SA, ^2^D_6_-JA and ^2^D_6_-JA-Ile, respectively), and then analyzed with high performance liquid chromatography/mass spectrometry following the method as described previously (Qi et al., 2016). To avoid individual differences, five replications were performed and each consisting of five individuals.

### *LsPDI1* Expression and Insect Feeding Performance

An experiment was designed using *S. frugiperda* and *M. persicae* on *N. benthamiana* hosts to determine the effects of *LsPDI1* expression on insect feeding preferences and offspring performance. LsPDI1 or GFP was transiently expressed in *N. benthamiana* for 24 h. Cell death induced by LsPDI1 appeared at 48 h post-infection. Target protein expression was observed under a Zeiss LSM750 confocal laser-scanning microscope (Carl Zeiss AG, Oberkochen, Germany). Leaves expressing LsPDI1 and GFP for 24 h were excised and placed pairwise in culture dishes. Eight second-instar *S. frugiperda* larvae or 15 newly emerged *M. persicae* female adults were placed in the center of each pair of leaves. Leaf selection by the insects was recorded at 12 and 24 h after feeding. Three biological replications were performed, and each comprised six leaf disks.

In the no-choice test, *M. persicae* fecundity assays were performed in 24-well plates according to Atamian et al. (2013), with some modification. Briefly, LsPDI1 or GFP was transiently expressed for 24 h in *N. benthamiana.* Cell death induced by LsPDI1 appeared at 48 h post-infection. Target protein expression was observed under a Zeiss LSM750 confocal laser-scanning microscope (Carl Zeiss AG, Oberkochen, Germany). Leaves expressing LsPDI1 and GFP for 24 h were excised and placed in a single 24-well plate. Four first-instar *M. persicae* nymphs were placed on each leaf disk. After 6 days, the nymphs were transferred to a new 24-well plate containing fresh leaf disks infiltrated 24 h earlier. On day 9, adult *M. persicae* were transferred to a new 24-well plate containing fresh leaf disks infiltrated 24 h earlier. On day 12, the adults were transferred to a new 24-well plate containing fresh leaf disks infiltrated 24 h earlier. Newly produced nymphs were recorded on days 9, 12, and 15. The average number of nymphs produced per adult was calculated by dividing the number of live adult aphids on days 9, 12, and 15. Three biological replications were performed and each comprised six leaf disks.

### Construction and Transformation of Recombinant Plasmid Containing *LsPDI1* Mutant

To explore the functional domain of *N. benthamiana* cell death induced by LsPDI1, seven *LsPDI1* mutants containing different domains were constructed and analyzed by *Agrobacterium*-mediated transient expression in *N. benthamiana* as described above. These primers were designed with Primer Premier v. 5.0 (Supplementary Table 1).

### Conservative Analysis of Salivary PDI1 Proteins Among Various Planthopper Species

The amino acid sequences of LsPDI1 and its orthologs NlPDI and SfPDI were aligned with DNAssist v. 2.2. The accession numbers for *NlPDI* and *SfPDI* are KU365961.1 and MF189030.1, respectively. The *NlPDI* and *SfPDI* coding regions were cloned, constructed into a *GFP* vector, and transiently transformed into *N. benthamiana* leaves as described above to detect cell death.

## Results

### LsPDI1 Is Secreted Into Rice During SBPH Feeding

**FIGURE 1:**
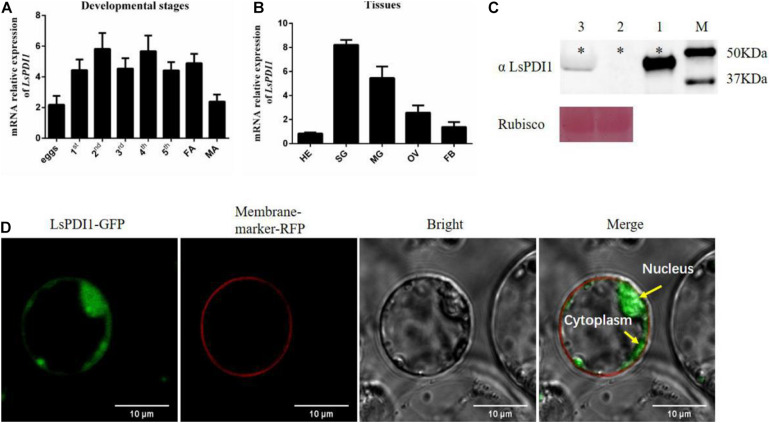
Spatiotemporal expression and subcellular localization of small brown planthopper (SBPH) LsPDI1 in rice protoplast. **(A,B)** *LsPDI1* expression at various developmental stages **(A)** and in different tissues **(B)** (*n* = 5). **(C)** Western blot detection of LsPDI1 protein (∼48 kDa) secreted in rice plants infested or uninfested by SBPH nymphs (*n* = 3). Lane 1, salivary glands extracts from SBPH fifth-instar nymphs. Lanes 2 and 3, extracts from rice plants either uninfested (Lane 2) or infested (Lane 3) with fifth-instar nymphs. M, molecular weight marker (kDa); ^∗^indicates the target band of protein; Rubisco was the loading sample control and Ponceau staining was used. **(D)** Transient LsPDI1 expression was localized to rice protoplast cytoplasm and nucleus. LsPDI1-GFP was expressed in rice protoplasts by polyethylene glycol-mediated transformation. Fluorescence signals were observed at 488 nm (GFP) and 562 nm (RFP) by confocal laser-scanning microscopy. RFP-marker acts as a membrane location indicator. Bar = 10 μm.

Transient expression-based salivary proteome screening identifies the salivary effectors implicated in plant-insect interactions. Here, we performed high-throughput, *Agrobacterium*-mediated transient salivary protein expression in *N. benthamiana.* It was based on the SBPH salivary proteome (Supplementary Table 2). We identified several important salivary effectors, including *LsPDI1*, which encodes a disulfide isomerase. We measured its spatiotemporal expression in various tissues and at different developmental stages. LsPDI1 was expressed during most of the developmental stages of SBPH (Figure 1A) and was highly expressed in the salivary gland and midgut (Figure 1B).

To confirm whether LsPDI1 is secreted into rice tissues during feeding, we extracted the proteins from uninfested leaf sheaths and from those infested with SBPH and performed western blotting on them. Figure 1C shows a band of ∼48 kDa in plants infected by SBPH (lane 3). The same band was also detected in extracts of SBPH salivary glands (lane 1). In contrast, no LsPDI1 band was detected in the uninfested control plants (lane 2). Therefore, we determined that LsPDI1 was transferred from SBPH salivary glands to the plants during feeding. Cellular localization after transient LsPDI1 expression in rice protoplasts showed that LsPDI1 was mainly located in the cytoplasm and nucleus (Figure 1D). Hence, LsPDI1 is secreted into rice tissue and might perform various functions in different organelles.

### LsPDI1 Induces ROS Burst and Cell Death in Plants

**FIGURE 2:**
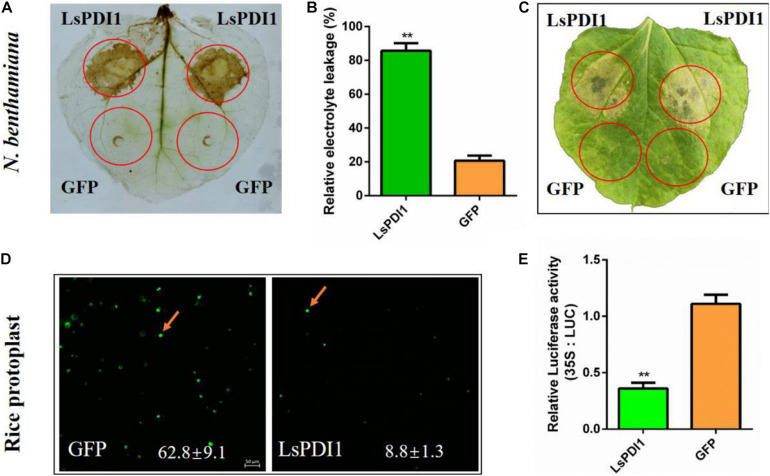
Transient LsPDI1 expression *in planta* induces reactive oxygen species (ROS) burst and cell death. **(A)** Detection of ROS burst by 3,3’-diaminobenzidine (DAB) staining after LsPDI1-GFP expressed transiently for 12 h. GFP was the negative control. **(B)** Detection of relative electrolyte leakage (%) after LsPDI1-GFP transiently expressed for 96 h. GFP was the negative control (*n* = 3). **(C)** *Nicotiana benthamiana* leaves were infiltrated with *Agrobacterium* carrying GFP and LsPDI1-GFP and photographed after agroinfiltration for 96 h. GFP was the negative control (*n* = 3). **(D)** Detection of living rice protoplasts by fluorescein diacetate (FDA) staining and confocal laser-scanning microscopy after transiently transforming mCherry or LsPDI1-mCherry for 16 h (*n* = 5). The mCherry was the negative control. Bar = 50 μm. **(E)** Detection of dual-luciferase (LUC) activity in rice protoplasts after transiently co-expressing LUC and LsPDI1 or LUC and GFP (negative control) for 40 h using an LUC assay system (*n* = 5). “**” indicates significant differences between treatments (*P* ≤ 0.01; Student’s *t*-test).

We transiently expressed LsPDI1 in *N. benthamiana* leaves with or without the signal peptide to elucidate the roles of this protein. Only LsPDI1 in the absence of signal peptide strongly induced cell death (Supplementary Figure 1), indicating it may be secretes into plant cells to play roles, but not in apoplasts. Furthermore, it induced both ROS burst and ion leakage. In contrast, GFP had no such effect (Figures 2A–C). We transiently expressed LsPDI1 in rice protoplasts in the absence of signal peptides and obtained similar results. FDA staining showed that the viability of protoplasts expressing LsPDI1 was markedly lower than that of control protoplasts expressing GFP (Figure 2D). Luciferase (LUC) activity in rice protoplasts co-expressing LsPDI1 and LUC was dramatically lower than that was in those co-expressing GFP and LUC in Figure 2E.

### LsPDI1-Mediated Cell Death Depends on a Calcium Signaling Pathway

**FIGURE 3:**
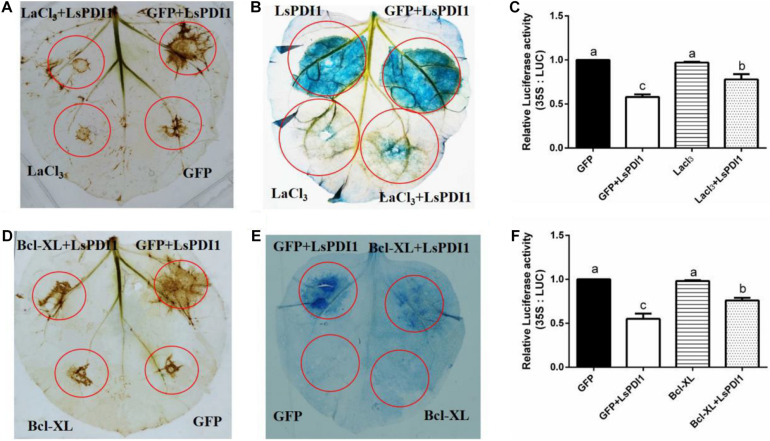
LaCl_3_ and Bcl-XL significantly inhibit cell death induced by LsPDI1 expression in plants. **(A,B)** Effects of the calcium ion inhibitor LaCl_3_ on plant reactive oxygen species (ROS) burst **(A)** and cell death **(B)** induced by transient LsPDI1 overexpression. The 3,3’-diaminobenzidine (DAB) or Trypan blue staining detection was developed after LsPDI1-GFP was expressed transiently for 12 or 48 h, respectively (*n* = 3). LaCl_3_ was added to resuspended *Agrobacterium* cultures bearing 1 mM plasmid for *Nicotiana benthamiana* leaf agroinfiltration. **(C,D)** Effects of anti-apoptosis protein Bcl-XL and LsPDI1 or GFP and LsPDI1 co-expression on ROS burst **(C)** and cell death **(D)**. DAB or Trypan blue staining detection was developed after LsPDI1-GFP was expressed transiently for 12 or 48 h, respectively (*n* = 3). *Nicotiana benthamiana* leaves were pre-infiltrated for 24 h with *Agrobacterium* cells bearing the *Bcl-XL* expression vector. **(E)** Effects of LaCl_3_ on relative LUC activity induced by transient *LsPDI1* expression in rice protoplasts (*n* = 6). An LUC assay system was developed after LsPDI1-GFP expressed transiently for 40 h. LaCl_3_ was added to rice protoplasts transfected with 1 mM plasmid. Lowercase letters indicate significant differences among treatments according to Duncan’s multiple range test (*P* ≤ 0.05). **(F)** Effects of Bcl-XL and LsPDI1 co-expression on relative LUC activity in rice protoplasts (*n* = 6). *Nicotiana benthamiana* leaves were pre-infiltrated for 24 h with *Agrobacterium* cells bearing the Bcl-XL expression vector. An LUC assay system was developed after LsPDI1-GFP expressed transiently for 40 h. Lowercase letters indicate significant differences among treatments according to Duncan’s multiple range test (*P* ≤ 0.05).

Calcium signaling plays a critical role in plant ROS burst and cell death (Dangl et al., 1996; Ma and Berkowitz, 2007; Durian et al., 2020). To determine whether calcium signaling is required for *LsPDI1*-induced cell death, we applied the calcium signaling inhibitor LaCl_3_ and the antiapoptotic protein Bcl-XL to *N. benthamiana* leaves and rice protoplasts. LaCl_3_ blocked ROS burst and cell death induced by transient LsPDI1 expression in *N. benthamiana* leaves (Figures 3A,B). LaCl_3_ enhanced LUC activity in rice protoplasts co-expressing LsPDI1 and LUC (Figure 3C). Transient LsPDI1 and Bcl-XL co-expression inhibited cell death more effectively than transient LsPDI1 and GFP co-expression (Figures 3D,E). LsPDI1, Bcl-XL, and LUC co-expression in rice protoplasts promoted LUC activity to a greater extent than LsPDI1, GFP, and LUC co-expression (Figure 3F). Therefore, LsPDI1-induced cell death may depend on a calcium signaling pathway.

### LsPDI1 Triggers Plant Defense Responses

**FIGURE 4:**
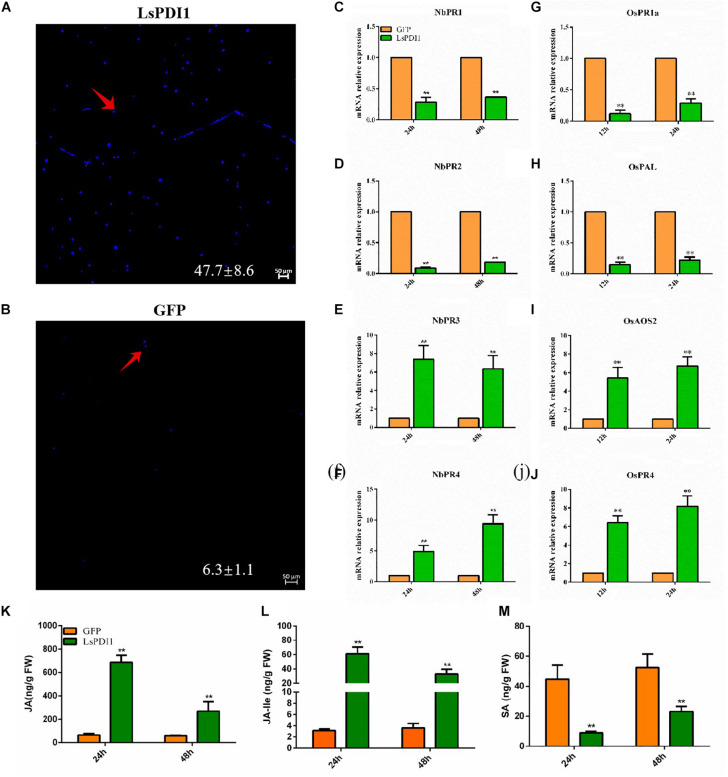
Transient LsPDI1 expression induces Jasmonic acid (JA)-mediated plant defense responses. **(A,B)** Detection of callose deposition induced by transient LsPDI1-GFP fusion protein expression in *Nicotiana benthamiana* leaves at 48 h (*n* = 6). GFP was used as the control. Callose deposition was detected by aniline blue staining. Bar = 50 μm. The red arrow indicates the spots of callose deposition. **(C–F)** Relative mRNA expression of *NbPR1* (salicylic acid [SA]) and *NbPR2* (SA), *NbPR3* (JA), and *NbPR4* (JA) induced by transient LsPDI1-GFP fusion protein expression in *Nicotiana benthamiana* leaves at 24 and 48 h (*n* = 5). **(G–J)** Relative mRNA expression of *OsPAL* (SA), *OsPR1a* (SA), *OsAOS2* (JA), and *OsPR4* (JA) induced by transient LsPDI1-GFP fusion protein expression in rice protoplast at 12 h and 24 h (*n* = 5). “**” indicates significant differences between treatments (*P* ≤ 0.01; Student’s *t*-test). **(K–M)** The JA **(K)**, JA-Ile **(L)**, and SA **(M)** hormone levels in the *Nicotiana benthamiana* leaves of transiently expressing LsPDI1 or GFP at 24 and 48 h (*n* = 5). “**” indicates significant differences between treatments (*P* ≤ 0.01; Student’s *t*-test).

To determine whether *LsPDI1* activates plant defense responses, we transiently expressed LsPDI1 in *N. benthamiana* leaves, evaluated callose deposition via aniline blue staining, and measured mRNA expression of the defense-related or pathogen resistance genes *NbPR1*, *NbPR2*, *NbPR3*, *NbPR4*, *OsPAL, OsPR1a, OsAOS2*, and *OsPR4* associated with JA and SA signaling pathways (Chaman et al., 2003; Luna et al., 2011; Tang et al., 2011; Liu et al., 2012). Aniline blue staining disclosed relatively more callose spots in leaves expressing LsPDI1 than in those expressing GFP. Thus, LsPDI1 markedly induces callose deposition (Figures 4A,B). RT-qPCR revealed that LsPDI1 dramatically induced mRNA expression of *NbPR3* and *NbPR4* (JA signaling pathway) but substantially repressed mRNA expression of *NbPR1* and *NbPR2* (SA signaling pathway) at 24 and 48 h (Figures 4C–F). In rice protoplasts, LsPDI1 considerably induced mRNA expression of *OsAOS2* and *OsPR4* (JA signaling pathway) but markedly repressed mRNA expression of *OsPAL* and *OsPR1a* (SA signaling pathway) compared with GFP at 12 and 24 h (Figures 4G–J).

Additionally, we measured SA, JA, and JA-Ile levels in transiently expressed LsPDI1 *N. benthamiana* leaves. Results showed that JA and JA-Ile levels in transiently expressed LsPDI1 *N. benthamiana* leaves were significantly higher than those in the control transiently expressed GFP *N. benthamiana* leaves at 24 and 48 h, whereas the SA levels were significantly decreased (Figures 4K–M). Taken together, LsPDI1 induces callose deposition and the JA signaling pathway but represses the SA signaling pathway.

### *In planta LsPDI1* Expression Decreases Insect Performance

**FIGURE 5:**
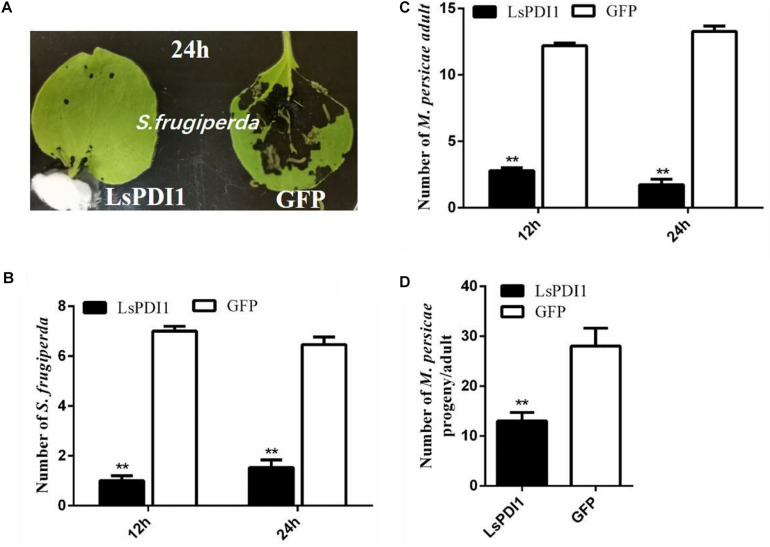
Transient LsPDI1 overexpression *in planta* decreases insect performance on host plant. **(A–C)** *Spodoptera frugiperda* or *Myzus persicae* have a greater preference for feeding on *Nicotiana benthamiana* leaves transiently overexpressing GFP than for those overexpressing LsPDI1. After 24 h of transient foliar LsPDI1 or GFP expression, eight second-instar *Spodoptera frugiperda* larvae or 15 newly emerged *Myzus persicae* were set in the middle of harvested leaves and the numbers of them on leaves were recorded at 12 and 24 h after feeding, respectively (*n* = 18). **(D)** Transient LsPDI1 overexpression decreased *Myzus persicae* fecundity on host plants. *Agroinfiltration* and aphid assays were performed side-by-side using LsPDI1-GFP and GFP. Average number of nymphs produced per adult is based on three replicates. Each replicate consisted of six replicated leaf disks (*n* = 18). “**” indicates significant difference between treatments (*P* ≤ 0.01, Student’s *t*-test).

LsPDI1 induces JA-mediated plant defense response. The JA signaling pathway plays key roles in plant defense responses against herbivorous insects. Therefore, LsPDI1 may attenuate host plant infestations by insects by promoting plant defense. Plant defense against insect infestation is often associated with non-preference (antixenosis) or decreased reproduction (antibiosis). Results of choice test showed that there were substantially fewer *S. frugiperda* or *M. persicae* on the rice leaves expressing LsPDI1 than on those expressing GFP at 12 and 24 h after feeding (Figures 5A–C). In the no-choice test, fewer nymphs per *M. persicae* adult were produced on leaves expressing LsPDI1 than on leaves expressing GFP (Figure 5D).

### The Functional Motif of LsPDI1 Is Located in Its *C*-Terminus

**FIGURE 6:**
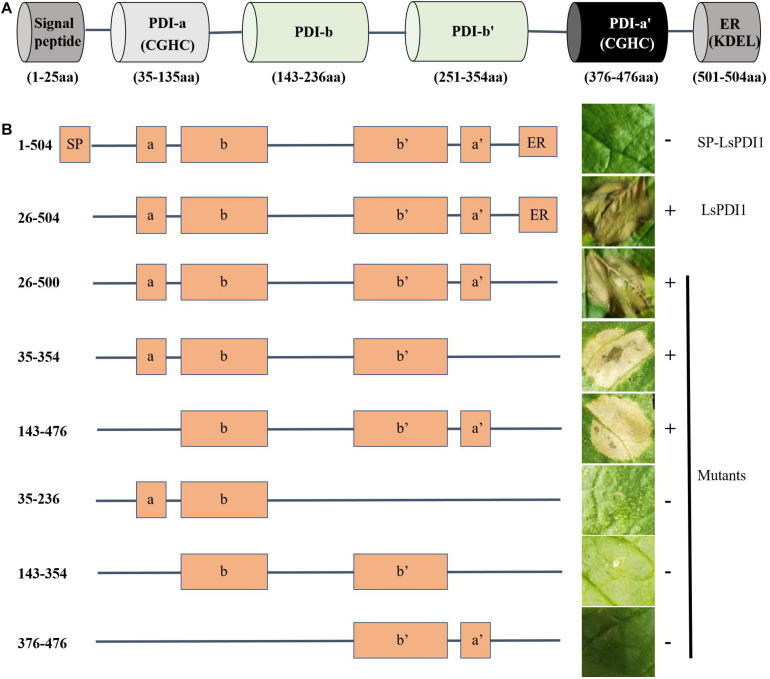
Determination of functional LsPDI1 domains causing cell death. **(A)** Functional domains of LsPDI1 have a *N*-terminal signal peptide for secretion, four thioredoxin domains (a, b, b’, and a’) predicted by an NCBI conserved domain search, and two catalytic domains containing characteristic CGHC active sites in the a and a’ domains. **(B)** Left panel indicates the amino acid positions for initiation and termination and schematic view of LsPDI1 deletion mutants. Right panel indicates the cell-death lesions on *Nicotiana benthamiana* leaves expressing *LsPDI1* deletion mutants. Symptoms were photographed at 72 h after infiltration (*n* = 3). SP indicates signal peptide. ER indicate retention signal of endoplasmic reticulum HEEL. “ + ” and “–” indicate presence and absence of cell death symptoms, respectively.

We searched the conserved domains of LsPDI1 using an NCBI conserved domain search software and analyzed its deletion mutants based on domain predictions to identify the functional motif responsible for cell death. We found four thioredoxin domains (a, b, b’, and a’) with two catalytic domains containing characteristic CGHC active sites in the a’ and a’ domains (Figure 6A). Transient expression assays revealed that mutants with the a and b, b and b’, or b and a’ domains could not induce cell death in *N. benthamiana* leaves. In contrast, the deletion mutants a-b-b’, b-b-a’, and a-b-b’-a’ could induce cell death. Thus, the *C*-terminal deletion domains a-b-b’ and b-b-a’ were required to enable *LsPDI1* to induce cell death (Figure 6B). Both the presence and absence of the *C*-terminal retention signal in the endoplasmic reticulum (ER) triggered cell death in *N. benthamiana* leaves. Overall, the domains a and a’ may be redundant in LsPDI1 and could be vital for activating plant defense.

### Salivary PDI1s in Three Planthopper Species Induce Plant ROS Burst and Cell Death

**FIGURE 7:**
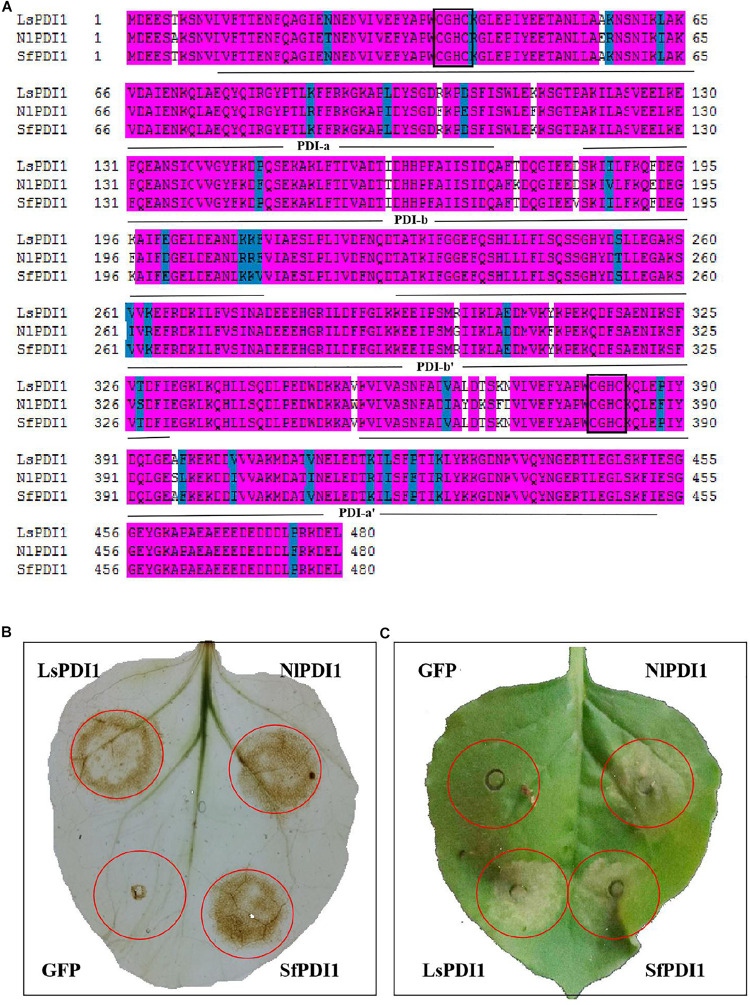
Determination of PDI1 conservation among the three planthopper species. **(A)** Alignment of amino acid sequences of LsPDI1 and its homologs based on the brown planthopper (BPH) and white-backed planthopper (WBPH) without the signal peptide. Identical amino acid residues are highlighted by pink ground shading. Similar amino acid residues are shaded with a light blue background. Different amino acids residues are not shaded. The four thioredoxin domains (a,b,b’, and a’) were indicated using an NCBI conserved domain search software. Active CGHC motif sequences are boxed in the a and a’ domains. **(B)** Determination of reactive oxygen species (ROS) burst symptoms caused by transiently expressing LsPDI1 and its homologs from BPH and WBPH in *Nicotiana benthamiana* (*n* = 6). GFP was the negative control. Symptoms were photographed at 12 h after infiltration. **(C)** Determination of cell death symptoms caused by transiently expressing LsPDI1 and its homologs from BPH and WBPH in *N. benthamiana* (*n* = 6). GFP was the negative control. Symptoms were photographed at 60 h after infiltration.

We searched the BPH and WBPH salivary proteome and genome databases to investigate PDI1 conservation across various planthopper species. We found that the sequences NlPDI and SfPDI were highly homologous with LsPDI1. Their amino acid sequences had 91.00 and 99.4% similarity, respectively (Figure 7A). For this reason, we determined that *LsPDI1* is highly conserved in all three planthopper species and may play similar crucial roles in all planthopper-plant interactions.

We transiently expressed NlPDI and SfPDI without the signal peptide in *N. benthamiana* leaves to confirm whether they also cause ROS burst and cell death. Results showed that expression of NlPDI and SfPDI significantly induced H_2_O_2_ accumulation and cell death in *N. benthamiana* leaves, whereas GFP did not (Figures 7B,C). Taken together, these results demonstrate that salivary *LsPDI1* orthologs are highly conserved across three planthopper species and all these genes strongly induce ROS burst and plant cell death.

## Discussion

There is a wealth of information on planthopper salivary proteins; nevertheless, little is known about their mechanisms in plant cells. Here, we discovered the novel salivary elicitor LsPDI1, which induces ROS burst, cell death, and the JA signaling pathway in plants. Seemingly, this mode of action may mediate the arms race between plants and herbivorous insects.

Cell death and ROS burst are critical plant defense responses (Wang et al., 2017; Ma et al., 2019). Cell death was strongly induced when LsPDI1 was transiently expressed in *N. benthamiana* leaves or rice protoplasts. Similar studies showed that salivary NlMLP or NlSP1 proteins in BPH induced host plant cell death (Shangguan et al., 2017; Huang et al., 2020). Thus, cell death could be a common strategy for plant defense response triggered by insect salivary protein. Early signaling events such as ROS production were strongly induced by transient LsPDI1 expression in *N. benthamiana* leaves and is positively correlated with cell death in transfected regions (Mur et al., 2008; Wang et al., 2017). Therefore, we speculate that excessive ROS production may be responsible for LsPDI1-induced plant cell death. ROS burst is an effective plant defense against herbivores, owing to its association with modulating phenolic compound metabolism and lignin biosynthesis in plant cell walls and could, therefore, inhibit herbivorous insect feeding (Gaquerel et al., 2015; Laitinen et al., 2017). A deletion mutant revealed that the *C*-terminal domains a-b-b’ or b-b-a’ are required to induce LsPDI1-mediated cell death in the absence of the signal peptide. A similar observation has been reported for a typical PDI gene in *P. parasitica* (Meng et al., 2015). LsPDI1-induced cell death was inhibited by LaCl_3_ and the antiapoptotic protein Bcl-XL associated with the calcium signaling pathway in *N. benthamiana* leaves and rice protoplasts. Hence, calcium signaling may regulate LsPDI1-induced cell death (Lecourieux et al., 2002; Boudsocq et al., 2010). In a previous study, LaCl_3_ and Bcl-XL inhibited plant cell death induced by the BPH salivary NlMLP (Shangguan et al., 2017).

The JA and SA signaling pathways play major roles in plant defense against herbivory (Gregg and Jander, 2008; Wu and Baldwin, 2010b; Su et al., 2019). Interestingly, it has been previously shown that they antagonistically regulate flg22-triggered oxidative bursts (Yi et al., 2014). Other similar studies demonstrated that plants preferentially regulate herbivorous insect resistance via interactions among phytohormone signaling pathways, including antagonistic crosstalk between SA and JA (Zarate et al., 2007; Thaler et al., 2012; Alon et al., 2013; Xu et al., 2018). Here, we measured the expression levels of key indicator genes related to SA and JA signaling, (Chaman et al., 2003; Liu et al., 2012; Kiba et al., 2014; Elodie et al., 2015; Ma et al., 2019) and the JA, JA-Ile, and SA hormone levels after *in planta* LsPDI1 expression. In *N. benthamiana*, LsPDI1 markedly upregulated *NbPR3* and *NbPR4* associated with JA but substantially downregulated *NbPR1* and *NbPR2* related to SA after 24 and 48 h. Similarly, in *N. benthamiana* LsPDI1 expression markedly induced JA and JA-Ile hormone levels but substantially suppressed SA hormone levels after 24 and 48 h. Thus, LsPDI1 positively influences JA-related signaling pathways and negatively influences SA-related signaling, respectively, and these effects were confirmed in rice protoplasts. Evidences have shown that JA signaling may protect host plants against chewing insects and certain piercing-sucking insects such as aphids and whiteflies (Kloth et al., 2016; Xu et al., 2018; Zhang et al., 2018; Chen et al., 2019). In accordance with this, *in planta* LsPDI1 expression enhanced plant defenses against chewing *S*. *frugiperda* and sap-sucking *M*. *persicae.* This may induce broad-spectrum insect resistance after salivary LsPDI1 was secreted into host plants. It is noteworthy that the transient LsPDI1 overexpression did not induce cell death at 12 or 24 h. Thus, cell death may not account for the observed results. These findings suggests that LsPDI1-induced plant defense against herbivorous insects is mediated by activation of the JA signaling pathway.

PDI1 orthologs are highly conserved across various planthopper species. After transient overexpression, PDI1 orthologs strongly induce ROS burst and plant cell death. Classical PDIs have also been detected in saliva proteomes of other piercing-sucking insects such as aphids (Carolan et al., 2011; Van Bel and Will, 2016) and spider mites (Zhu et al., 2018). For this reason, PDIs may have a common interaction mechanism between various herbivorous insects and their plant hosts and may be able to induce broad-spectrum insect resistance. Therefore, LsPDI1 has potential application prospects for pest control as a plant vaccine in pest control.

In summary, the present study revealed LsPDI1 as a novel salivary elicitor inducing plant defense responses. *In planta* LsPDI1 expression may initially induce calcium signaling as an early defense mechanism. Subsequently, downstream signaling pathways related to ROS burst and JA are activated and host plant resistance to insects is strengthened. These discoveries may elucidate the molecular mechanism of plant-insect interactions and lay the foundation for the development of innovative management strategies to control insect pest infestations. ROS burst, JA activation and cell death as defenses against a broad range of plant insect infections, and we found *in planta* LsPDI1 expression enhanced plant defenses against chewing *S*. *frugiperda* and sap-sucking *M. persicae*. Therefore, we predicted that LsPDI1 may enhance plant defenses against SBPH. However, further research should be conducted using transgenic *LsPDI1* rice plants to determine its insect resistance against planthopper. Moreover, future research should also focus on determine whether and where LsPDI1 was really secreted into plant cells using *in situ* immunofluorescence of SBPH-infested rice leaf sheath, identifying host plant receptors of herbivorous insect salivary LsPDI1 using a yeast two-hybrid assay, and cultivating overexpression and knockout transgenic rice lines of target receptor protein; and thus to reveal how a conserved PDI could induce ROS burst, cell death, and immune responses in plants.

## Data Availability Statement

The original contributions presented in the study are included in the article/Supplementary Material, further inquiries can be directed to the corresponding authors.

## Author Contributions

JFu, JFa, and RJ designed and performed the experiments and wrote the manuscript. YS analyzed the data. LW, HZ, and JL helped revise the manuscript. All authors contributed to the article and approved the submitted version.

## Conflict of Interest

The authors declare that the research was conducted in the absence of any commercial or financial relationships that could be construed as a potential conflict of interest.
