# Supplementary material for: Planthopper-Secreted Salivary Disulfide Isomerase Activates Immune Responses in Plants
Source: Front Plant Sci. 2021 Jan 18;11:622513. doi: 10.3389/fpls.2020.622513 (PMC7848103; doi:10.3389/fpls.2020.622513)
Supplement: Supplementary file 1 [file Data_Sheet_1.docx]

**Supplementary information**


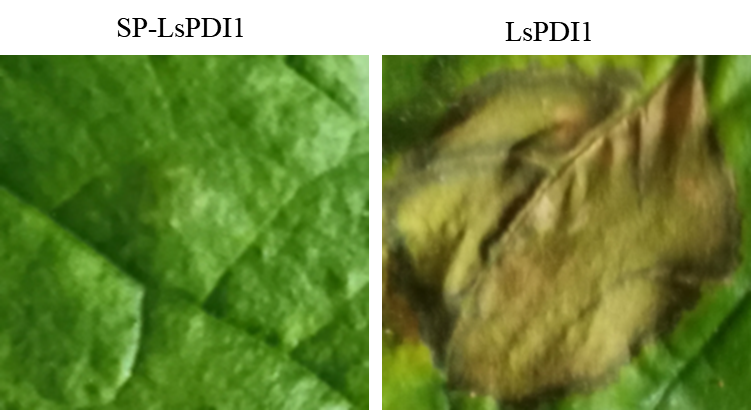


**Figure S1** Transient *LsPDI1* expression without signal peptide in planta induces plant cell death. SP-LsPDI1 indicates *LsPDI1* containing signal peptide. The *N. benthamiana* leaves were infiltrated with *Agrobacterium* carrying *SP-LsPDI1-GFP* or LsPDI1-GFP and photographed after agroinfiltration for 96 h (n=6).

**Table S1 Primers used in this study.**

| **Primer** | **Sequences (5’-3’)** |
| --- | --- |
| *LsPDI1-GFP-SP-F* | CGGGGTCGACGGATCCATGTTTATGGATTTCACCGAAATGT |
| *LsPDI1-GFP-SP-R* | TGCTCACCATGGATCCCAACTCGTCCTTTCTCGGAAGG |
| *LsPDI1-GFP-F* | CGGGGTCGACGGATCCATGGATGAAGAATCAACCAAAAGTA |
| *LsPDI1-GFP-R* | TGCTCACCATGGATCCCAACTCGTCCTTTCTCGGAAGG |
| *Bcl-XL-GFP-F* | CGGGGTCGACGGATCCATGTCTCAGAGCAACCGGGAG |
| *Bcl-XL-GFP-R* | TGCTCACCATGGATCCTTTCCGACTGAAGAGTGAGC |
| *LsPDI1-mCherry-F* | CGGGGTCGACGGATCCATGGATGAAGAATCAACCAAAAGTA |
| *LsPDI1-mCherry-R* | TGCTCACCATGGATCCCAACTCGTCCTTTCTCGGAAGG |
| *NlPDI1-GFP-F* | CGGGGTCGACGGATCCATGGACGAGGAGAGCGCC |
| *NlPDI1-GFP-R* | TGCTCACCATGGATCCCAGCTCGTCCTTCCTGAACAGG |
| *SfPDI1-GFP-F* | CGGGGTCGACGGATCCATGGATGAAGAATCAACCAAAAGTA |
| *SfPDI1-GFP-R* | TGCTCACCATGGATCCCAACTCGTCCTTTCTCGGAAGG |
| **Primers used in qPCR** |  |
| *LsPDI1-F* | AAACTTTGCCGACGTTGCTC |
| *LsPDI1-R* | CCTTGAAAGCTTCGCCCAAC |
| *NbPR1-F* | CCTTCATTTCTTCTTGTCTC |
| *NbPR1-R* | AGGTTACAATCTGCAGCCAA |
| *NbPR2-F* | CAATGCATTAGCAGCAGCAG |
| *NbPR2-R* | ATCTTTGGGCGGGTAGGTAT |
| *NbPR3-F* | TGGGGTTATTGCTGGCTTAG |
| *NbPR3-R* | GGGTCATCCAAAACCAGAGA |
| *NbPR4-F* | GGCCAAGATTCCTGTGGTAGAT |
| *NbPR4-F* | CACTGTTGTTTGAGTTCCTGTTCCT |
| *OsPAL*-F | AGCACATCTTGGAGGGAAGCT |
| *OsPAL*-R | GCGCGGATAACCTCAATTTG |
| *OsAOS2*-F | CAATACGTGTACTGGTCGAATGG |
| *OsAOS2*-R | AAGGTGTCGTACCGGAGGAA |
| *OsPR1a*-F | GTGGACCCGCACAACGCG |
| *OsPR1a*-R | GCCGATCGCCGTCGAGTC |
| *OsPR4*-F | ACCCACAACAGAACAACTG |
| *OsPR4*-R | CCAACCTCTTCCATCTTATTG |
| *Nbsactin*-F | CTGGGTTTGCTGGAGATGAT |
| *Nbactin*-R | CATCTCCCACGTAGGCATCT |
| *NbGAPDH -F* | GGTGCCAAGAAGGTTGTGAT |
| *NbGAPDH -R* | TAGTGCAACTGGCATTGGAG |
| *Lsactin*-F | AATCGTAAGAGACATCAAGGAG |
| *Lsactin*-R | AGGCAATTCGTAGGACTTCT |

The underscore indicates the joint of the primer for homologous recombination.

**Table S2.**  Salivary proteins of *L. striatellus* identiﬁed by LC-MS/MS

| **Protein classification** | **Description** | **SignalP** | **Uni Pep** |
| --- | --- | --- | --- |
| **Digestive enzymes and hydrolases** |  |  |  |
| **Oxidoreductases** | *Glyceraldehyde-3-phosphate dehydrogenase, GAPDH* | **N** | **5** |
|  | Hydroxyacid oxidase 1-like, HAO 1 | N | 2 |
|  | Superoxide dismutase, SOD | N | 3 |
|  | Ferredoxin-NADP oxidoreductase（FNR） | N | 2 |
| **Transferase** | Phosphoglycerate kinase 1 | N | 5 |
|  | Hydroxymethyltransferase | N | 3 |
| **Hydrolases** |  |  |  |
| Esterase | GDSL-like Lipase/Acylhydrolase | Y | 2 |
|  | *Carboxylesterase, CES* | **Y** | **5** |
| Nuclease | plancitoxin-1 | Y | 4 |
|  | *Placental protein 11, PP11* | **Y** | **8** |
| Glycohydrolase | Maltase 2-like | Y | 2 |
|  | Beta-hexosaminidase subunit beta-like, Hex B-like | **Y** | **6** |
|  | Alpha-L-fucosidase-like, AFU | **Y** | **7** |
|  | Beta-glucuronidase, GUS | **Y** | **8** |
|  | *Alpha-N-acetylgalactosaminidase-like, NAGA* | **Y** | **11** |
| Protease | *Trypsin 9* | **Y** | **5** |
|  | *Trypsin 26* | **Y** | **5** |
|  | *Stubble-2* | **Y** | **11** |
|  | Putative serine protease K12H4.7 | Y | 3 |
|  | putative serine protease F56F10.1 | Y | 3 |
|  | Dipeptidyl peptidase 4, DPP4 | **Y** | **19** |
|  | Venom dipeptidyl peptidase 4（VDDP-4） | N | 8 |
|  | Venom dipeptidyl peptidase 4-like (VDDP-4 like) | N | 9 |
|  | Cathepsin B-like protease, CB-like | **Y** | **12** |
|  | Leucyl aminopeptidase | N | 2 |
|  | Homologue of sarcophaga 26, 29 kDa proteinase | Y | 2 |
|  | Plasma glutamate carboxypeptidase, PGCP | **Y** | **6** |
|  | Lysosomal aspartic protease, LAP | **Y** | **6** |
|  | ATP-dependent zinc metalloprotease FtsH-like | N | 2 |
|  | Matrix metalloproteinase-24, MMP-24 | N | 2 |
|  | Aminopeptidase N | Y | 6 |
|  | Aminopeptidase N-like isoform X2, APN-like isoform X2 | N | 5 |
|  | Endothelin-converting enzyme 1-like, ECE 1 | **Y** | **6** |
| Anhydride hydrolase | Elongation factor Tu, EF-Tu | N | 2 |
|  | Elongation factor 1-alpha, EF-1α | **Y** | **12** |
| Lyase (synthetase) | Fructose-1,6-diphosphate aldolase | N | 2 |
|  | Carbonic anhydrase 7（CA7-like） | **Y** | **7** |
|  | Ribose-1,5-diphosphate carboxylase / oxygenase subunit (Rubisco) | N | 2 |
|  | Enolase | **Y** | **5** |
| Isomerase | Triosephosphate isomerase | N | 2 |
|  | Triosephosphate isomerase-like | N | 2 |
|  | **protein disulfide-isomerase (LsPDI1)** | Y | 2 |
| Ligase | Glutamine synthetase | N | 2 |
| **Calcium binding protein** | Annexin A7, ANX | **Y** | **7** |
|  | Annexin B10-like1 | **Y** | **7** |
|  | Annexin B10-like2 | **Y** | **8** |
|  | Annexin-like protein RJ4 | Y | 9 |
|  | *Calmodulin-like, CaM-like* | **N** | **11** |
|  | Regucalcin-like, RGN-like | **Y** | **6** |
|  | EF-hand motif protein | **Y** | **9** |
|  | Synaptotagmin-4-like, Syt 4 | **Y** | **5** |
| **Transport protein** | Lipophorin precursor, LP | **Y** | **12** |
|  | Apolipoprotein D | Y | 2 |
|  | Apolipoprotein D-like | Y | 2 |
|  | Conserved oligomeric Golgi complex subunit 4(COG 4) | N | 2 |
| **Salivary sheath protein** | Mucin-like protein, MLP | Y | 8 |
|  | Mucin-17 | **Y** | **6** |
|  | Mucin-19 | **Y** | **6** |
| **Cytoskeleton associated protein** | Beta- actin | N | 2 |
|  | Tropomyosin 1 | N | 2 |
|  | Dynein intermediate chain 3, ciliary, putative | Y | 2 |
|  | Lamin Dm0 | N | 4 |
| **Nucleotide and chromatin binding protein** | Heat shock protein 70 (Hsp 70) | N | 3 |
|  | Adenine nucleotide translocator, ANT | N | 2 |
|  | Polyubiquitin-like protein | N | 2 |
|  | Histone H2A.V | N | 2 |
|  | Histone H3.3-like protein | N | 2 |
| **Proteins associated with photosystem II (PSII)** | Photosystem II oxygen evolving enhancer protein 1（OEE1） | N | 4 |
|  | Cytochrome b6-f complex iron-sulfur subunit | N | 2 |
|  | Photosystem II D1 protein | N | 3 |
|  | Photosystem II 43 kDa protein | N | 2 |
|  | Light-harvesting complex protein LHCG11 | N | 2 |
| **Other proteins** | Vitellogenin | Y | 3 |
|  | Rootletin | **Y** | **8** |
|  | 14-3-3 protein epsilon-like | N | 2 |
|  | 14-3-3 zeta-like | N | 2 |

The blackbody indicate target gene for the present study. “Y” indicates “Yes”, “N” indicates “No”.
